# Enhanced Living by Assessing Voice Pathology Using a Co-Occurrence Matrix

**DOI:** 10.3390/s17020267

**Published:** 2017-01-29

**Authors:** Ghulam Muhammad, Mohammed F. Alhamid, M. Shamim Hossain, Ahmad S. Almogren, Athanasios V. Vasilakos

**Affiliations:** 1 Department of Computer Engineering, College of Computer and Information Sciences, King Saud University, Riyadh 11543, Saudi Arabia; 2 Department of Software Engineering, College of Computer and Information Sciences, King Saud University, Riyadh 11543, Saudi Arabia; mohalhamid@ksu.edu.sa (M.F.A.); mshossain@ksu.edu.sa (M.S.H.); 3 Department of Computer Science, College of Computer and Information Sciences, King Saud University, Riyadh 11543, Saudi Arabia; ahalmogren@ksu.edu.sa; 4 Department of Computer Science, Electrical and Space Engineering, Lulea University of Technology, 93187 Skellefteå, Sweden; athanasios.vasilakos@ltu.se

**Keywords:** voice pathology assessment, enhanced living environment, Saarbrucken voice database, co-occurrence matrix, Gaussian mixture model

## Abstract

A large number of the population around the world suffers from various disabilities. Disabilities affect not only children but also adults of different professions. Smart technology can assist the disabled population and lead to a comfortable life in an enhanced living environment (ELE). In this paper, we propose an effective voice pathology assessment system that works in a smart home framework. The proposed system takes input from various sensors, and processes the acquired voice signals and electroglottography (EGG) signals. Co-occurrence matrices in different directions and neighborhoods from the spectrograms of these signals were obtained. Several features such as energy, entropy, contrast, and homogeneity from these matrices were calculated and fed into a Gaussian mixture model-based classifier. Experiments were performed with a publicly available database, namely, the Saarbrucken voice database. The results demonstrate the feasibility of the proposed system in light of its high accuracy and speed. The proposed system can be extended to assess other disabilities in an ELE.

## 1. Introduction

The number of people in the world suffering from different pathologies has increased for several reasons, including pollution, excessive abuse of certain organs, and stress. These pathologies hinder the normal lives of people; however, the introduction of smart homes makes these lives easier in many senses. In smart homes, smart solutions incorporating machine intelligence or artificial intelligence are embedded [1]. One of the major applications of these smart solutions is healthcare [2]. Some of the healthcare solutions in smart homes have been reported in the literature. A platform for behavior monitoring of diabetes patients was proposed in [3]. A summary work on the development of smart homes in relation to the rehabilitation of neurologically disabled patients can be found in [4]. Smart home solutions for elderly people have been proposed and highlighted in [5,6,7].

### 1.1. Motivation

Though there are a number of smart solutions for different pathologies, very few exist for voice pathology assessment. In one study, it was found that more than 7.5% of the total population in America suffer from voice pathology [8]. Singers and teachers suffer the most from voice disorders, because they use their voice excessively. It has been reported that around 20% of American teachers have voice pathologies [8]. Most teachers with these pathologies feel shy or do not have time to visit medical doctors or specialized clinics for treatment or assessment. Even other people with voice disorders do not go to therapists for rehabilitation for many reasons such as residing in remote places, traffic congestion, and the difficulties of the appointment. Therefore, there is a need for smart solutions to assess voice pathologies in a smart home framework, where the patients do not need to physically go to the doctor or to clinics. This will be an added feature of an enhanced living environment (ELE).

Voice pathology detection or assessment research can be divided into two categories: subjective and objective. The subjective assessment requires trained doctors and special equipment, which are very costly. Moreover, sometimes this assessment varies from doctor to doctor depending on the experience or perception. The objective assessment does not need special equipment, and the result is always unbiased if the algorithm is correct; however, we should also mention that the objective assessment can only assist a medical doctor, or can only be used for primary screening. The final decision should come from medical doctors.

### 1.2. Related Work

Most of the existing voice pathology detection or assessment systems use features from either speech or speaker recognition applications or voice quality measurement [9]. The features include Mel-frequency cepstral coefficients (MFCCs) [10], linear prediction coefficients (LPCs), shimmer, jitter, harmonic to noise ratio [11], and MPEG audio features. Recently, features from image processing applications have been used in voice pathology detection systems. For example, an interlaced derivative pattern (IDP) was used in [12], and fractal dimension was used in [13]. These image-feature-based detection systems achieved good accuracy; however, they need to be carefully chosen after some feature selection or feature reduction techniques. Some nonlinear dynamics-based features were also used, but the accuracy was not very good [14].

A voice contour of the voice signal was used to discriminate between normal and pathological samples in [15], because pathologies in the vocal folds cause the voice to weaken and fluctuate. This method may not be successful if the recording of the voice is done in a different environment or by a diverse microphone. In [16], the authors used an information theory-based technique, called correntropy, to distinguish between normal and pathological voices. The accuracy was around 97%.

Wavelet decomposition has been used in several voice pathology detection methods. For example, Saidi and Almasganj used M-Band wavelets and found optimal parameters of wavelets using a genetic algorithm [17]. Fractal dimensions from different wavelet subbands were used in [13]. The latter study claims that the low-frequency band is useful for differentiating between a normal and a pathological voice. A wavelet packet transform- and singular value decomposition-based method was proposed in [18]. Different classifiers were used in the method, and a reasonable accuracy was achieved.

Zhong et al. proposed a vocal fold damage detection method using a type-2 fuzzy classifier, where the input voice was transformed by a short-time Fourier transform and a singular value decomposition [19]. The whole method was deployed in a heterogeneous sensor network.

### 1.3. Contribution

In this paper, we propose a smart solution to assess voice pathologies using co-occurrence matrices and a Gaussian mixture model (GMM). Co-occurrence matrices were primarily designed to extract texture features of images and were proved successful for texture classification [20]. This is a computationally efficient yet powerful feature extraction technique. We take advantage of co-occurrence matrices to design a voice pathology assessment system. In the proposed system, two types of inputs are used: voice signal and electroglottography (EGG) signal. Smart phones are used as sensors to capturing voice signals, and EGG electrodes are used as sensors to capture EGG signals. These two signals are processed separately and later combined after the individual classification stage. The experiments were performed on a publicly available database, namely, the Saarbrucken voice database (SVD) [21]. The experimental results demonstrate the efficiency of the proposed system.

There are two major contributions of the paper: (i) introducing multi-sensors as inputs to the voice pathology assessment system; and (ii) applying co-occurrence matrices to extract features from voice and EGG signals.

## 2. Materials and Methods

In the experiments, we used data from the SVD. The SVD is a large collection of voice and EGG signals from normal people and people with different voice pathologies. While recording, speakers were asked to speak sustained /a/, /i/, and /u/ at high, low, and medium pitch level. All speakers were native Germans. The recording was done in a controlled office environment. There were many types of voice pathologies among half of the speakers; these pathologies included vocal fold polyp, sulcus, vocal fold nodules, vocal fold cyst(s), and vocal fold paralysis (unilateral or bilateral). Not all the speakers’ inputs are used in the current study; we chose only those speakers who had both voice signals and EGG signals. There were approximately 400 such speakers. We selected signals when the speakers uttered sustained /a/.

**Figure 1:**
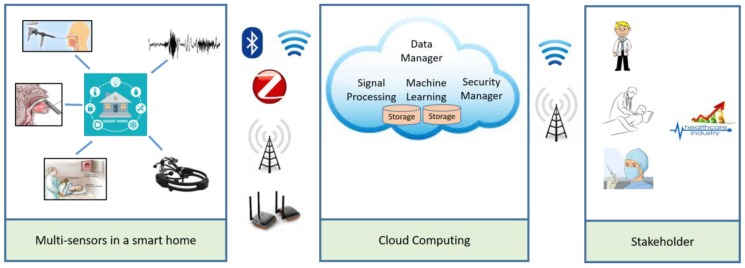
A framework of the voice pathology assessment using the cloud.

An overall framework of the proposed system in a cloud environment is shown in Figure 1. There are three main components, which are smart home, cloud computing, and stakeholders. The smart home is equipped with many sensors such as a stroboscope, a laryngoscope, smart phones, voice recorders, and an EGG machine. In our case, only smart phones and the EGG machine were used. These devices capture voice and EGG signals, respectively, from the patients at the home. The captured signals are then transmitted to the cloud via the Internet for processing and classification. In the cloud, the main components are a security manager, which checks the authenticity of the users, a data manager, which distributes data to the appropriate dedicated servers, a signal processing unit, which extracts features, and a machine learning unit, which classifies the signals into normal or pathological. The decision of the system along with the decision score is sent to some registered doctors or nurses, who then make the final decision. According to the decision, the patient is notified and prescribed some therapy, which can be given in the patient’s home.

It can be noted that the framework was realized in the proposed system using only certain aspects. For example, in the “Multi-sensors in a smart home” block, we used smart phones for voice signals and the EGG machine for EGG signals. The signals are then transmitted via a broadband connection to the cloud. The cloud component service was set up conferring to the Web Services Resource Framework (WSRF) [22]. In particular, we used Amazon Web Services and a SQL server.

**Figure 2:**
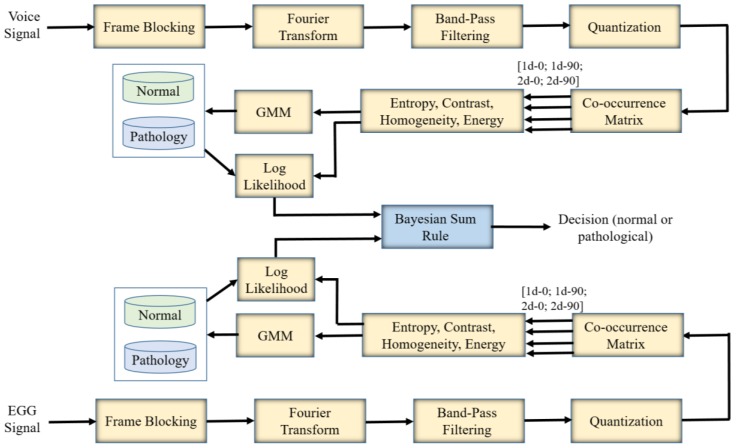
Block diagram of the proposed voice pathology system using the voice signal and the EGG signal.

Figure 2 shows the block diagram of the proposed voice assessment system. There are two inputs to the system: the voice signal and the EGG signal. The processing and classification steps are described below.

Processing Steps:

- Divide the voice signal into 40 ms non-overlapping frames. This is referred to “Frame Blocking” in the figure.
- Apply Fourier transform to each of the blocks to convert the time-domain signal into the frequency-domain signal.
- Apply 24 band-pass filters to the frequency-domain representation. The center frequencies of the filters are spaced on Mel scale between 200 Hz and 8000 Hz. The successive filters are overlapped by 4 octaves. It is expected that the energy at the lower frequencies is more concentrated for a normal voice than that for a pathological voice.
- Quantize the energy values of the filters to eight levels ranging from 1 to 256. This quantization is required for the co-occurrence matrices. The frames are arranged in sequence, and an image (similar to a Mel-spectrogram) is formed.
- Apply co-occurrence matrices on the image in two directions (0° and 90°) and two distances (immediate neighborhood and the second neighborhood). Then, we have four co-occurrence matrices. The co-occurrence matrices are calculated on a 12 (frame) × 24 (filter) window.
- Calculate energy, entropy, contrast, and homogeneity features from these matrices. Therefore, we have 16 features per window. These features are then fed into the GMM-based classifier.

The same processing steps are applied to the EGG signal as well.

Classification Steps:

- A five-fold cross-validation approach is used.
- For each iteration, using the training dataset, one GMM for the normal voice and one GMM for the pathological voice are created using the features obtained from the processing steps. The number of Gaussian mixtures are varied to find the optimal one.
- In the same iteration, the features of the test subset are compared with the GMMs and the log-likelihood scores for the normal case and for the pathological case are obtained.
- Using the Bayesian Sum Rule [23], we fuse the scores from the voice signal and the EGG signal, and find the final decision.
- The decision along the scores is then sent to the stakeholders.

Co-Occurrence Matrix:

This matrix is calculated in the processing step. If the image (Im) size is *A* × *B*, the co-occurrence matrix, *COOCCUR*, can be found by the following equation:

COOCCURx,y=∑u=1A∑v=1B(Imu,v=x)∧(Imu′,v′=y)

where *x* and *y* are the energy levels of filters. *u*’ and *v*’ are calculated by the following equation.

$$\begin{array}{l} u^{\prime }=u+d\cos\phi \\ v^{\prime }=v+d\sin\phi \end{array}|d\in \{1,2,3,...,N\},\phi \in \{0{}^{\circ},90{}^{\circ},180{}^{\circ},270{}^{\circ}\}$$.

In the above equation, the value of *N* can take any integer number not exceeding the boundary of the image. The choice of φ is such that the direction is only rectangle. For example, if we choose d = 2, and φ = 90°, then we have $u^{\prime }=u+2\times 0=u\;\mathrm{and}\;v^{\prime }=v+2\times 1=v+2$. It means that the compared locations have a distance between them “*distance 2 straight down*.”

**Figure 3:**
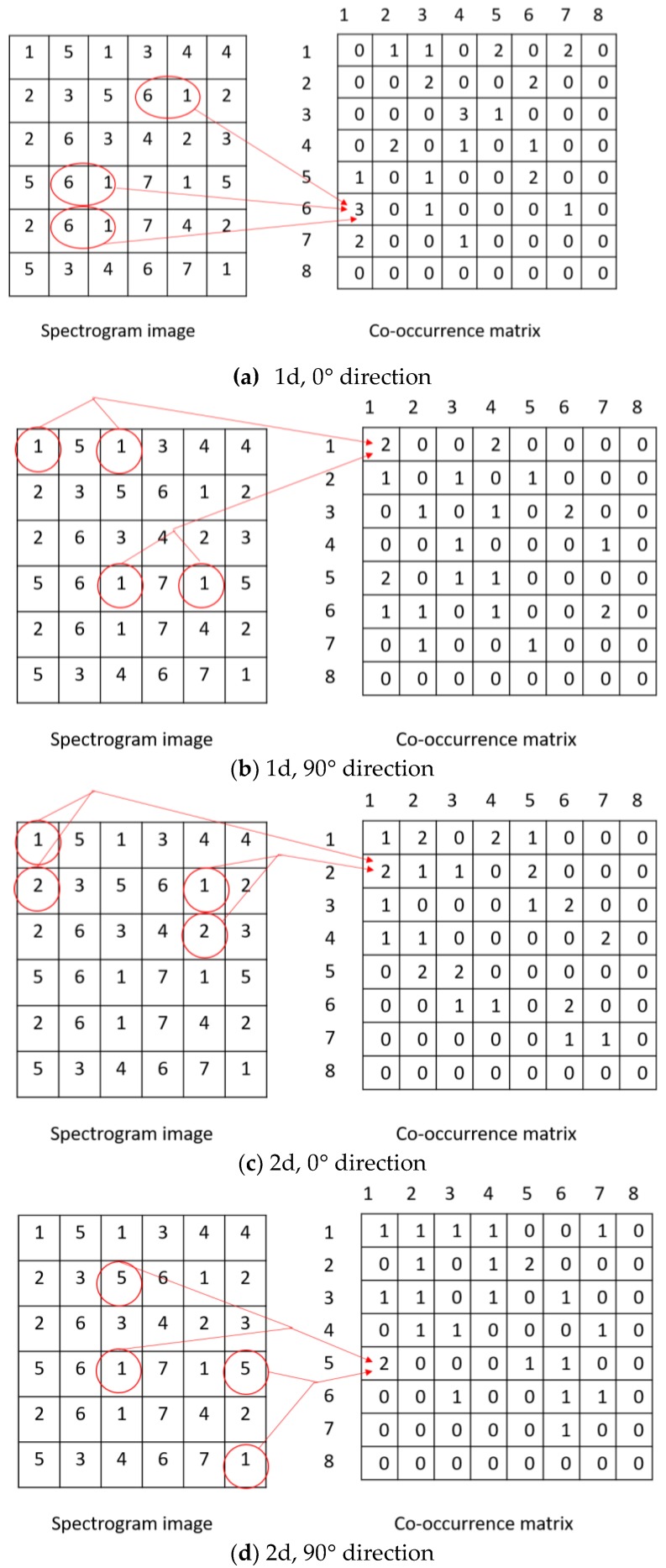
Illustration of calculating co-occurrence matrices in (**a**) 1d and 0° direction; (**b**) 1d and 90° direction; (**c**) 2d and 0° direction; and (**d**) 2d and 90° direction.

In our proposed system, we used two values of d (d = 1, 2) and two values of φ (φ = 0°, 90°). The purpose of d = 1 is to compare the energy levels of two immediate neighborhoods, while that of d = 2 is to compare those of two second-nearest neighborhoods. We use both these values to extract multiresolution features from the signals. The two values of φ represent a relationship in time axis and frequency axis, respectively. An illustration of the matrix calculation is shown in Figure 3.

Four features are extracted from each of the matrices. These features are energy, entropy, contrast, and homogeneity. The calculation of the features is as follows:

$$\begin{array}{l} Energy=\sum_{z=1}^{A}\sum_{z^{\prime }=1}^{B}{\left[COOCCUR(z,z^{\prime })\right]}^{2}\\ Entropy=\sum_{z=1}^{A}\sum_{z^{\prime }=1}^{B}COOCCUR(z,z^{\prime })\ln[COOCCUR(z,z^{\prime })]\\ Contrast=\sum_{z=1}^{A}\sum_{z^{\prime }=1}^{B}\left[{\left(z-z^{\prime }\right)}^{2}]COOCCUR(z,z^{\prime }\right)\\ Homogeneity=\sum_{z=1}^{A}\sum_{z^{\prime }=1}^{B}\frac{COOCCUR(z,z^{\prime })}{1+{\left(z-z^{\prime }\right)}^{2}} \end{array}$$.

## 3. Results

**Figure 4:**
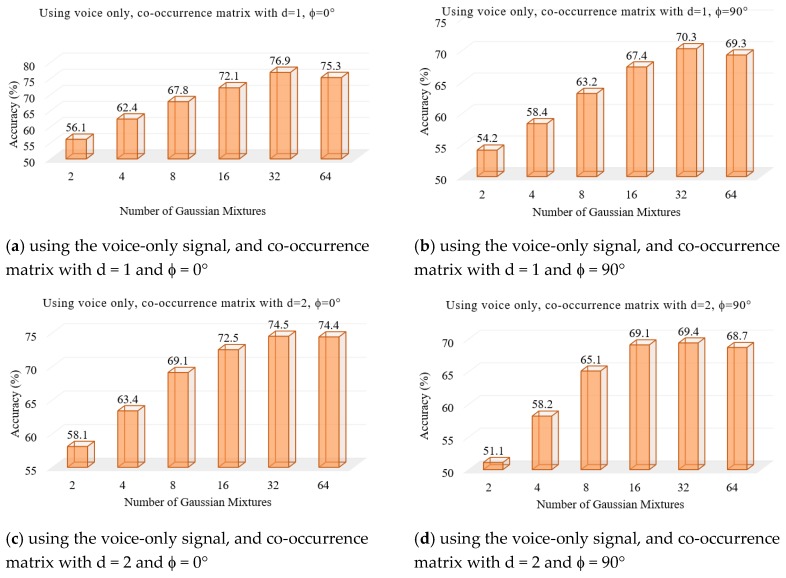
Accuracy of the system using the voice-only signal in different numbers of Gaussian mixtures.

**Figure 5:**
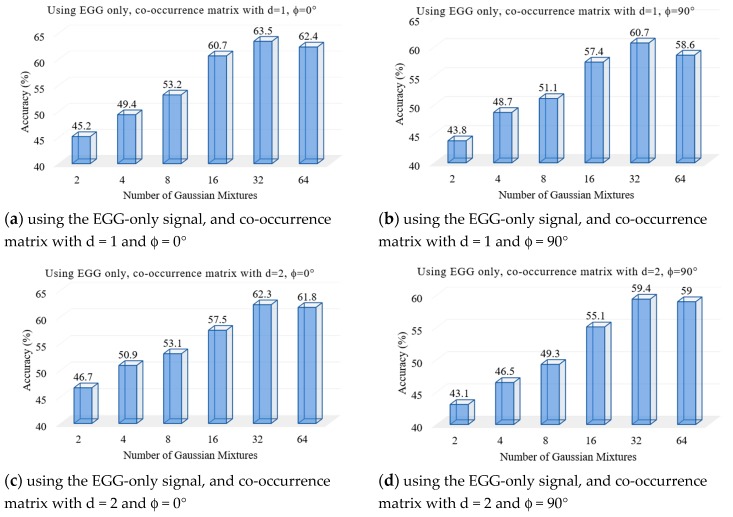
Accuracy of the system using the EGG-only signal in different numbers of Gaussian mixtures.

We performed a set of experiments to test the proposed voice pathology assessment system. First, two experiments were to find the optimal number of Gaussian mixtures using the voice signal and the EGG signal, separately. Figure 4 and Figure 5 show the results, respectively. From these figures, we find that 32 mixtures achieved the best results for both signals.

**Figure 6:**
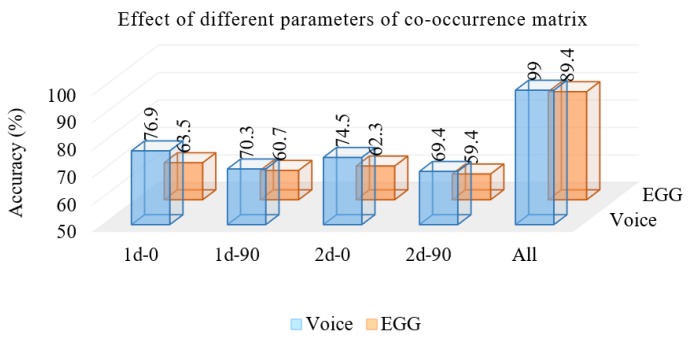
Accuracy of the system using features from different co-occurrence matrices.

The next experiment was to see the effect of different co-occurrence matrices on the system. We used 32 mixtures of the GMM. Figure 6 shows the accuracies of the system using the experiment. From the figure, we see that d = 1 (1d) achieved better accuracy than d = 2 (2d), and the horizontal direction achieved better accuracy than the vertical direction. A similar trend was observed for both signals. Using all four matrices, the system with the voice-only signal achieved 99% accuracy, while that with the EGG-only signal, 89.4% accuracy. From this outcome, we understand that the voice pathology can be assessed more accurately by the voice-only signal than by the EGG-only signal.

When we combined the likelihood scores using the Bayesian Sum Rule, the accuracy of the system moved to 99.87%, which is quite high comparing to other related systems. The average time taken by the system was 1.45 s per patient. This time included the processing time and the classification time.

**Figure 7:**
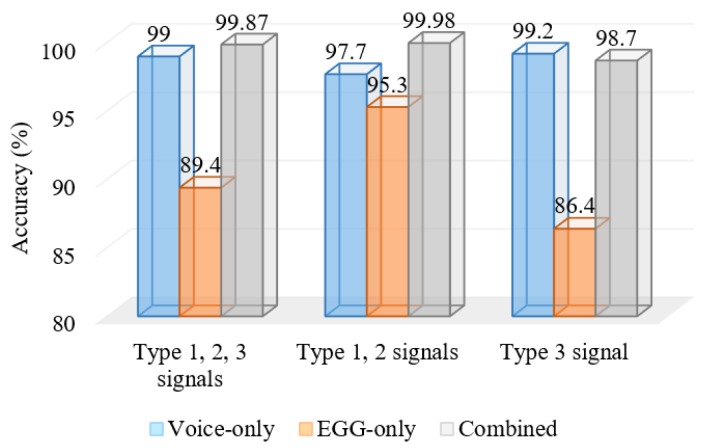
Accuracies of the proposed systems in different “Type” signals.

Titze classified voiced signals into three types: Type 1, Type 2 and Type 3 [24]. Type 1 signals are periodic or nearly periodic, Type 2 signals have strong modulations, while Type 3 signals are irregular and aperiodic. In the case of mild voice pathology, the voiced signal is either Type 1 or Type 2. A voice from a severe voice pathology is of Type 3. The acoustic analysis in most of the cases yielded a good result for Type 1 and Type 2 signals. In the next set of experiments, we wanted to see how the voice-only signal and the EGG-only signal fare in Types 1 and 2, and in Type 3. Out of the 400 samples chosen in the previous experiments, we found that 70 of them were of Type 3, and the rest were of either Type 1 or Type 2. Figure 7 shows the accuracies of the proposed system in Type 1 and 2, Type 3 only, and Types 1, 2 and 3. From the figure, we see that the voice-only signal performed far superior than the EGG-only signal in Type 3 (severe voice pathology). In mild to moderate voice pathologies (Types 1 and 2), the EGG-only signal performed very well; moreover, in this case, the combined voice and EGG signals performed the best (99.98%).

**Figure 8:**
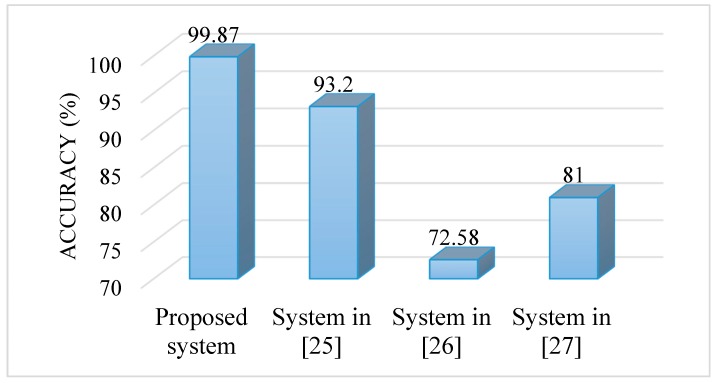
Accuracies of different systems.

We compared the proposed system with other related systems. The systems that we considered for comparison were proposed in [25,26,27]. These systems did not use both the signals; rather, they used only the voice signal. The system in [25] used the IDP features, that in [26] utilized the multi-dimensional voice parameters, and that in [27] used the MFCC features. Figure 8 shows the accuracies of the systems. From the figure, we see that the proposed system outperformed other compared systems. All of these systems were evaluated on the SVD. The accuracies of the other systems were obtained from the respective papers.

## 4. Discussion

A voice pathology assessment system has been proposed using two types of signals, voice and EGG. These two signals are obtained through two different types of sensors. Each of these signals is processed in the cloud. Co-occurrence matrices are obtained from the frequency-domain representation of the signals. Four features are calculated from each of these matrices. Two GMM-based classifiers, one each for voice-only and EGG-only signals, are used as the classification. The use of the co-occurrence matrix and the subsequent features are new in voice pathology assessment systems. Moreover, integrating two types of signals in such systems is not frequent. We successfully designed the system using these novel attributes.

With the rapid growth of Internet of Things, the smart home has become more user-friendly and usable to the masses. We designed a voice pathology assessment system using inputs from multi-sensors for an ELE. Our system is easy to use for patients because of the non-invasive nature of the sensors. Even if one sensor is not working, the system can work with other sensors.

There are several voice pathology detection systems in the literature; however, all of these systems work on a single signal. For example, systems in [10,11,13,25,26,27] use the voice signal as input. Sometimes, voice signals cannot guarantee high accuracy, which depends on the severity of the pathology. A person with a less severe voice pathology can pronounce the sustained vowel will ease; in this case, the voice will sound more like a voice from a normal person. A complementary type of signal, such as an EGG signal, can solve this problem to some extent, as we have seen from the results shown in Figure 7. EGG electrodes sense the movement of the vocal folds, and they are not mixed with vocal tract functions. On the other hand, EGG electrodes sometimes miss the minute movements of vocal folds. This can be improved by a sophisticated design of electrodes, but this is beyond the scope of the paper.

The system time requirement can be improved by deploying dedicated servers or units in the cloud for the two different signals. In this way, the processing of these signals will be in parallel, thereby reducing the time needed. One of the advantages of the proposed system is that it uses the same type of processing for both signals, unlike the system described in [28].

## 5. Conclusions

A voice pathology assessment system using voice and EGG signals is here proposed. To extract features from these signals, energy, entropy, contrast, and homogeneity are calculated from the co-occurrence matrices obtained from the spectrograms of the signals. The classifiers’ scores from each signal are fused by using the Bayesian sum rule to obtain the final decision whether the signal is normal or has a voice pathology. From the experiments, we arrive at certain conclusions, which are (i) for severe voice pathology, the decision from the EGG-only signal may not be accurate; (ii) for mild voice pathology, the EGG-only signal is a good competitor with the voice-only signal for accuracy; and (iii) in general, the combination of the voice-only signal and the EGG-only signal improves the accuracy of the voice pathology assessment system.

The future direction of this research can be how to integrate other Internet of Things to assess voice pathology. Furthermore, another input modality in the form of the non-invasive high-speed camera can be utilized within the system.
